# Zinc Acetate Hydrate Supplementation versus Polaprezinc Supplementation for Improving Hypozincemia in Hemodialysis Patients: A Randomized Clinical Trial

**DOI:** 10.1155/2023/2403755

**Published:** 2023-10-05

**Authors:** Etsuko Kumagai, Kazuhiro Furumachi, Akihiro Kurihara, Ken Hosokawa, Keiko Hosohata, Shinji Takai

**Affiliations:** ^1^Kenwakai Hospital, 1936, Kanaenakadaira, Iida, Nagano 395-0801, Japan; ^2^Department of Innovative Medicine, Osaka Medical College, 2-7, Daigaku-cho, Takatsuki, Osaka 569-8686, Japan; ^3^Japanese Red Cross Society Shimoina Red Cross Hospital, 3159-1, Motoojima, Matsukawa-machi, Shimoina-gun, Nagano 399-3303, Japan; ^4^Shimoina Kosei Hospital, 481-13, Yoshida, Takamori-machi, Shimoina-gun, Nagano 399-3102, Japan; ^5^Education and Research Center for Clinical Pharmacy, Osaka University of Pharmaceutical Sciences, 4-20-1, Nasahara, Takatsuki, Osaka 569-1094, Japan

## Abstract

Zinc supplementation may ameliorate zinc deficiency in maintenance hemodialysis patients; however, no standard protocol has been established. This study aimed to investigate the effects of zinc acetate hydrate (ZAH) and polaprezinc (PPZ) as zinc supplements in hemodialysis patients. We enrolled 75 hemodialysis patients with serum zinc levels <60 *μ*g/dL for this study and randomly assigned Zinc supplementation to these 75 patients: 37 received ZAH (50 mg/day), and 38 received PPZ (34 mg/day). Serum zinc levels of both groups were compared every 4 weeks for 1 year. In both groups, serum zinc levels significantly increased at 4–52 weeks. Serum zinc levels were significantly higher in the ZAH group at 4–12 weeks; however, no significant differences were observed between the groups at 16–52 weeks. Adverse events requiring a reduction in the zinc dose, including copper deficiency, occurred significantly more frequently in the ZAH group. In conclusion, PPZ can safely maintain serum zinc levels for 1 year. ZAH provides rapid zinc supplementation but can cause adverse events.

## 1. Introduction

Zinc deficiency is common in individuals with kidney failure, kidney transplantation, anorexia, malnutrition, and older individuals. Zinc deficiency is caused by decreased dietary intake, intestinal malabsorption, and loss into dialysate or urine. Malnutrition, inflammation complex, erythropoietin-resistant anemia, and atherosclerosis are renal-specific zinc-deficient diseases [1]. Japan's Practical Guidelines for Zinc Deficiency 2018 by the Japanese Society of Clinical Nutrition recommends zinc supplementation in patients with symptoms of zinc deficiency and hypozincemia (serum zinc level <60 *μ*g/dL) and confirmation of improvement in symptoms with zinc supplementation in various pathological conditions, including renal disease [2]. However, the Kidney Disease Outcomes Quality Initiative Clinical Practice Guidelines for Nutrition in chronic kidney disease (CKD): 2020 update states “There are no specific guidelines for monitoring zinc deficiency or supplementation in CKD. Future research is recommended to evaluate whether the same thresholds as in the general population apply to patients with CKD” [3].

Zinc supplementation increases serum zinc levels and ameliorates various symptoms associated with zinc deficiency in maintenance hemodialysis (HD) patients. However, zinc supplementation duration varied widely in previous studies. Wang et al. reviewed 15 randomized controlled trials on zinc supplementation and found that the intervention period in four studies was 42 days, 60 days in four studies, 90 days in five studies, 112 days in one study, and 360 days in one study [4].

This study aimed to evaluate the effect and safety of 1-year zinc supplementation with zinc acetate hydrate (ZAH) or polaprezinc (PPZ) in maintenance HD patients. ZAH and PPZ are covered by Japanese health insurance as hypozincemia treatment.

## 2. Materials and Methods

### 2.1. Research Design

This was a prospective, multicenter, active-controlled, randomized, open-label, parallel-group, comparative study based on written informed consent. Registration was open from December 1, 2019, to May 31, 2020. The participants included patients aged 40–94 years at the time of consent, were undergoing HD therapy thrice weekly, and had a dialysis history of ≥2 years.

This study was approved by the Kenwakai Hospital Ethics Committee (approval number 201920) and conducted in accordance with the Declaration of Helsinki. The study was registered with the University Hospital Medical Information Network (UMIN 000038759). Written informed consent was obtained from all the patients.

### 2.2. Patient Selection

**Figure 1:**
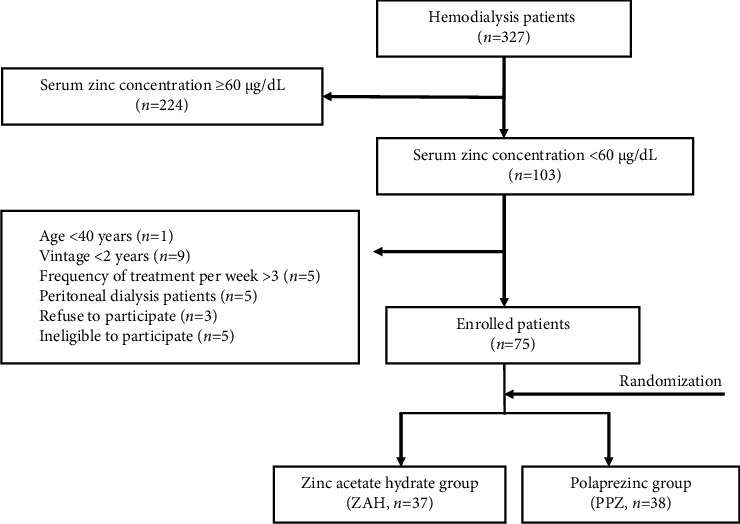
Patient selection and randomization. Serum zinc levels are measured in 327 patients on maintenance HD who undergo HD sessions more than thrice weekly in one of the three facilities. Of the 327 patients, 224 patients with zinc levels ≥60 *μ*g/dL were excluded. Of the 103 patients with zinc levels <60 *μ*g/dL, 28 patients were excluded due to lack of eligibility based on the exclusion criteria. The remaining 75 HD patients are allocated to the zinc acetate hydrate (ZAH) group or the polaprezinc (PPZ) group in a 1 : 1 randomization ratio. We finally allocated 37 and 38 patients to the ZAH and PPZ groups.

We evaluated 327 maintenance HD patients based on blood test results. Of the 327 patients, 224 patients with serum zinc levels ≥60 *μ*g/dL were initially excluded. Furthermore, 28 patients who did not meet the following inclusion criteria were excluded: age ≥40 years, HD vintage ≥2 years, frequency of HD treatment ≥3 times per week, not receiving peritoneal dialysis, acceptance for participation, and eligibility for participation. Seventy-five patients finally remained and were randomized in a 1 : 1 ratio to receive either ZAH (ZAH group, *n* = 37) or PPZ (PPZ group, *n* = 38) following the UMIN medical research support/case registration system (Figure 1).

### 2.3. Sample Size Calculation

The number of patients was determined by comparing the percentage of patients receiving ZAH and PPZ who achieved the target serum zinc concentration (>80 *μ*g) 1 month from baseline. A previous study on HD patients showed that ZAH increased serum zinc concentration at 1 month, with a 64% target achievement rate. The PPZ target achievement rate is 23% [5]. Based on the data, the difference in the arithmetic mean between the ZAH and PPZ groups was 40%, and their standard deviation was 20%, assuming that the variances of the two groups were the same. When using EZR, version 1.35, the number of cases necessary for conducting a two-tailed test with a significance level of *α* = 0.05 and a power of 80% is 27. The target number of cases was set at 30 in each group, for 60 cases, assuming that 5% of patients dropped out due to copper deficiency or gastrointestinal symptoms as major adverse responses, died, or were untraceable.

### 2.4. Supplementation Protocol

The starting doses of zinc supplementation were 50 and 34 mg of elemental zinc daily in the ZAH and PPZ groups. A ZAH tablet containing 25 mg of zinc was administered orally twice daily in the ZAH group, whereas a polaprezinc tablet containing 17 mg of elemental zinc was administered twice daily in the PPZ group. With a target of serum zinc levels of 80–130 *μ*g/dL, ZAH was titrated within a dose range of 25–50 mg/dL of zinc at each observation point, while polaprezinc was titrated within a dose range of 17–34 mg/dL of elemental zinc. These two drugs were administered within the scope described in each package insert. Supplementation was withheld in cases with two consecutive serum zinc levels >200 *μ*g/dL or two consecutive serum copper levels <30 *μ*g/dL, symptomatic hypocupremia, or drug-related adverse events.

Serum zinc and copper levels were monitored every 4 weeks until 24 weeks after the initial administration and again at 52 weeks. The mean values of serum zinc concentration, the proportion of patients with a serum zinc concentration of ≥80 *μ*g/dL, and the mean values of serum copper concentration were compared between the groups at baseline and every observation point. In addition, the incidence of symptoms suggestive of the side effects of therapy, such as gastric discomfort, vomiting, diarrhea, constipation, pruritus, eczema, sleeplessness, and anorexia, was investigated and compared between the two groups at each observation point.

### 2.5. Measurement

Blood samples were collected before the first HD session of the week in which monitoring for this study was planned. Serum zinc levels were measured using automatic enzyme immunoassay device JCA-ZS050 (JEOL, Tokyo, Japan) with Accuras Auto Zn (Shino-Test Corporation, Sagamihara, Japan). Serum copper levels were measured using automatic enzyme immunoassay device JCA-BM9130 (JEOL, Tokyo, Japan) with Quick Auto Neo Cu (Shino-Test Corporation, Sagamihara, Japan).

### 2.6. Statistical Analysis

All data were presented as the mean ± SD. For statistical processing, multiple comparisons for quantitative variables were performed using the Wilcoxon test and Dunnett's test, while the *χ*-square test and binary logistic regression were used for qualitative variables. In both analyses, the significance level was set at *p* < 0.05. All statistical analyses were performed using JMP Version 13.2 (SAS Institute Inc., Cary, NC, USA).

## 3. Results and Discussion

### 3.1. Patient Characteristics

The average ages of the ZAH and PPZ groups were 74.7 years and 72.6 years, respectively.  Table 1 shows the background patient characteristics according to groups. The baseline mean serum zinc and copper concentrations in the ZAH group were 52.3 *μ*g/dL and 89.1 *μ*g/dL, respectively, while those in the PPZ group were 52.4 *μ*g/dL and 93.5 *μ*g/dL, respectively.

### 3.2. Changes in Serum Zinc Concentration

**Figure 2:**
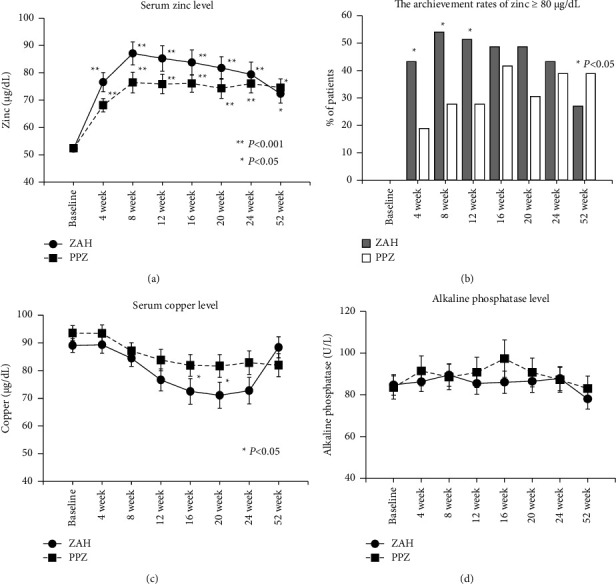
Effects of zinc supplementation on trace elements. (a) The mean serum zinc levels are significantly increased from baseline to all the observation points in both the ZAH and PPZ groups. (b) The achievement rate of a zinc level ≥80 *μ*g/dL is significantly higher in the ZAH group than in the PPZ group at 4–12 weeks. (c) The mean serum copper levels in the ZAH group decreased significantly from baseline to 16 weeks and 20 weeks, while those in the PPZ group did not significantly change during the observation period. (d) The mean alkaline phosphatase levels did not change in either group during the observation period.

Figure 2(a) shows the mean serum zinc concentration changes in the two groups. In the ZAH group, the mean serum zinc concentration was significantly increased from 52.3 *μ*g/dL at baseline to 77.5 *μ*g/dL 4 weeks after the start of oral ZAH administration, to 86.5 *μ*g/dL at 24 weeks, and further to 83.4 *μ*g/dL at 52 weeks (*p* < 0.001). Similarly, in the PPZ group, the mean serum zinc concentration was also significantly increased at every observation point from 52.4 *μ*g/dL at baseline to 68.1 *μ*g/dL at 4 weeks, 80.8 *μ*g/dL at 24 weeks, and 79.9 *μ*g/dL at 52 weeks (*p* < 0.001). The mean serum zinc concentration was significantly higher in the ZAH group than in the PPZ group at 4–12 weeks after the start of oral zinc administration (*p* < 0.05). However, no significant between-group difference was observed at 16 weeks after the start of supplementation.  Figure 2(b) shows the transition of the proportion of patients with serum zinc concentrations of ≥80 *μ*g/dL. The proportions of patients with serum zinc levels ≥80 *μ*g/dL at 4, 8, and 12 weeks (45.7%, 64.5%, and 65.5%, respectively) were significantly higher in the ZAH group than those in the PPZ group. However, no significant differences were observed in the proportions of such patients between the two groups at 24 and 52 weeks.

### 3.3. Changes in Serum Copper Concentrations

Figure 2(c) shows the mean serum copper concentration changes in the two groups. The mean serum copper concentration in the ZAH group decreased significantly from baseline (89.1 *μ*g/dL) to 70.6 *μ*g/dL at 16 weeks and 69.5 *μ*g/dL at 20 weeks (*p* < 0.05), recovering after that to 76.8 *μ*g/dL at 24 weeks and 83.6 *μ*g/dL at 52 weeks. Similarly, the mean serum Cu concentration in the PPZ group decreased significantly from baseline at 16 and 52 weeks (*p* < 0.05). No significant differences were observed in the mean copper concentrations between the two groups at any observation point.  Figure 2(d) shows the changes in the mean serum alkaline phosphatase concentration, a zinc-dependent enzyme, between the two groups. No significant differences between the two groups were observed from baseline to any of the observation points in either group. In addition, no significant changes were observed during the observation period for parameters other than serum zinc and copper levels in either group (Supplemental Material).

### 3.4. Occurrence of Adverse Events

Table 2 shows the incidence of adverse events requiring a reduced zinc dose or discontinuation during the observation period. Most adverse events were observed within 24 weeks from the start of oral zinc administration as follows: increase in serum zinc concentration to ≥130 *μ*g/dL in 11 (14.7%) patients; decrease in serum copper concentration to <30 *μ*g/dL in 6 (8.0%) patients; gastrointestinal-related symptoms, such as gastric discomfort, vomiting, diarrhea, and constipation in 39 (52.0%) patients; blood-related symptoms, such as anemia and thrombocytopenia in 11 (14.7%) patients; skin-related symptoms, such as pruritus, eczema, and rash in 4 (5.3%) patients; and other symptoms, such as sleeplessness and anorexia in 3 (4.0%) patients (Table 2(A)).

**Figure 3:**
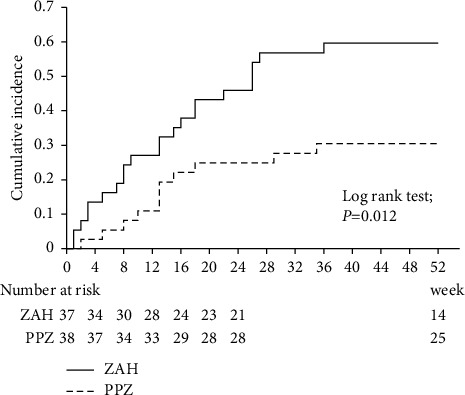
Kaplan–Meier curves comparing the occurrence transitions of zinc dose reduction or discontinuation of oral administration between the ZAH and the PPZ groups. Adverse events occurred significantly earlier and more frequently in the ZAH group than in the PPZ group throughout the observation period (*p* = 0.012).

In addition, between 24 weeks and 52 weeks, two patients developed a decrease in serum copper concentration to <30 *μ*g/dL (2.7%), and one patient developed other symptoms (1.3%) (Table 2(B)). No significant between-group differences were observed in the incidence of adverse events within 24 and 52 weeks. However, a significant difference was observed when all the adverse events requiring zinc dose reduction or discontinuation were combined.  Figure 3 compares the occurrence of zinc dose reduction or discontinuation between the ZAH and PPZ groups. Adverse events occurred earlier and were significantly more frequent in the ZAH group than in the PPZ group throughout the observation period (*p* = 0.012).

### 3.5. Changes in the Number of Patients Who Continue to Receive Zinc and the Mean Zinc Dose Administered

**Figure 4:**
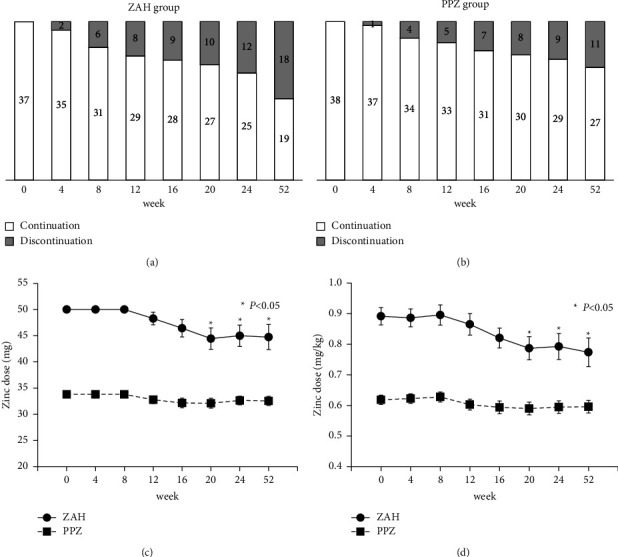
Changes in the number of patients who continued zinc supplementation and in the mean zinc dose in the ZAH and PPZ groups. (a, b) Changes in the number of patients who continued zinc supplementation versus those who had discontinued zinc supplementation at each observation point in the ZAH group (a) and the PPZ group (b). (c) Changes in the mean zinc dose administered in the ZAH and PPZ groups based on the number of patients who continued zinc supplementation with either the initial dose or the reduced dose. (d) Changes in the mean administered dose per kg body weight in the ZAH and PPZ groups. In the ZAH group, the average dose after 20 weeks is 44.44 mg, which is significantly lower than the initial dose (50 mg). Meanwhile, there are no significant changes in the drug dose in the PPZ group. Zinc administration is reduced or discontinued more frequently in the ZAH group than in the PPZ group throughout the study period.

Figures 4(a) and 4(b) show changes in the proportion of patients in the ZAH and PPZ groups who continued or discontinued zinc supplementation at each observation point.  Figure 4(c) shows the mean administered zinc dose variation in the ZAH and PPZ groups based on the number of patients who continued zinc supplementation with the initial or reduced doses. After 20 weeks, the mean dose in the ZAH group was 44.44 mg, significantly lower than the initial dose (50 mg). In the PPZ group, no significant changes in the drug dose were observed. The two-line graphs in Figure 4(d) show the evaluation of the changes in the mean dose administered per kg body weight, which is similar to the mean zinc dose administered, and has almost the same shapes as in Figure 4(c). Zinc administration was reduced or discontinued more frequently in the ZAH group than in the PPZ group throughout the study period.

### 3.6. Changes in the Hemoglobin (Hb) Level, Erythropoiesis-Stimulating Agent (ESA) Dosage, and Erythropoietin Resistance Index (ERI)

**Figure 5:**
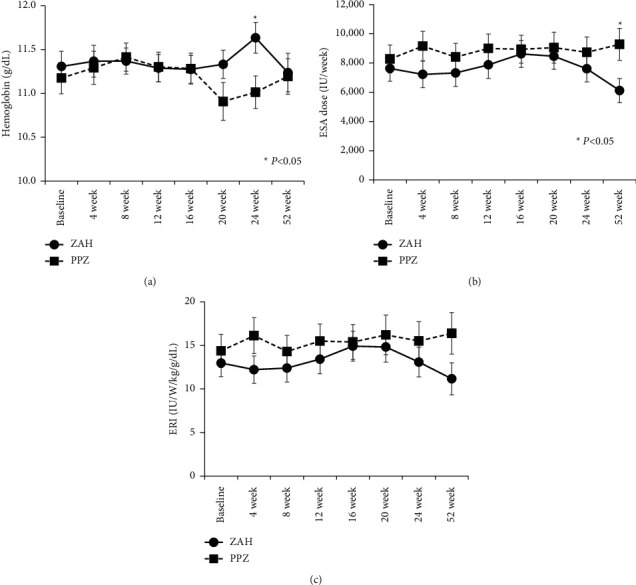
Changes in the hemoglobin (Hb) level, erythropoiesis stimulating agent (ESA) dosage, and erythropoietin resistance index (ERI). The Hb level remains within 11.2–11.6 g/dL in the ZAH group and within 10.9–11.4 g/dL in the PPZ group until week 52, with significant differences between the two groups only at week 24. ESA use is consistently lower in the ZAH group than in the PPZ group during the observation period. However, no significant between-group difference was observed in ESA use except at 52 weeks. The ERIs of both groups show line graphs similar to their respective ESAs but were not significantly different throughout the observation period. (a) Hemoglobin level, (b) ESA dosage, (c) ERI.

Figure 5 shows the changes in indicators related to anemia. Hemoglobin (Hb) remained within 11.2–11.6 g/dL in the ZAH group and within 10.9–11.4 g/dL in the PPZ group until week 52, with significant differences between the two groups only at week 24. The administration of erythropoiesis-stimulating agents (ESAs) was consistently lower in the ZAH group than in the PPZ group during the observation period, except at week 52. The erythropoietin resistance indices (ERIs) of both groups presented line graphs similar to their respective ESAs but were not significantly different throughout the observation period.

### 3.7. Correlations between Serum Zinc Concentration and Other Parameters

**Figure 6:**
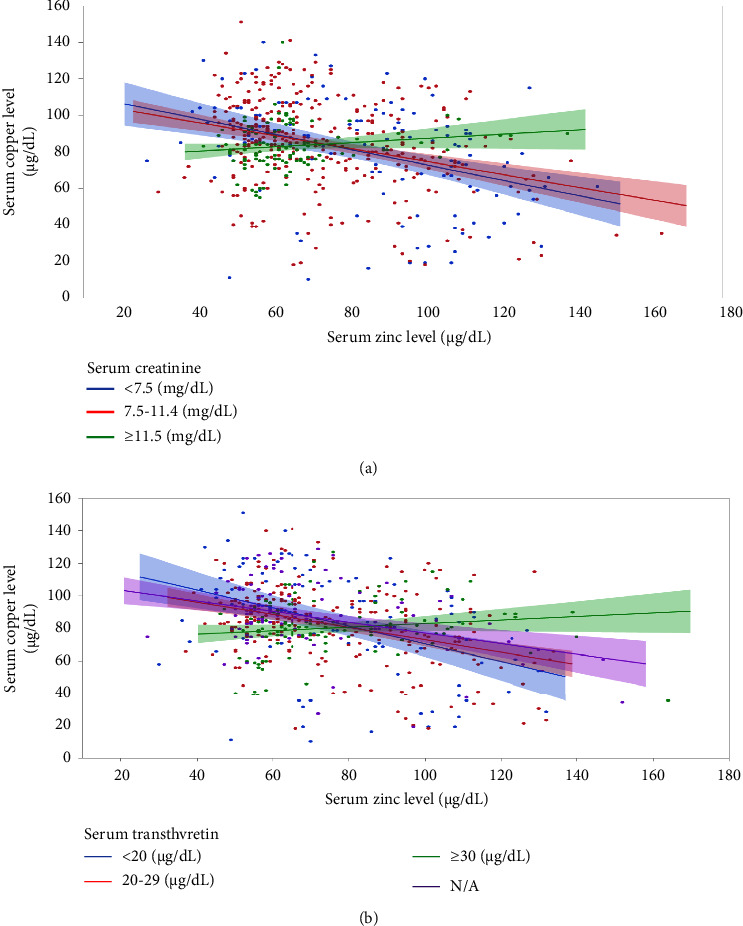
Scatter plots showing the correlation between serum zinc concentration and serum copper concentration. (a) With regression lines reflecting serum creatinine levels stratified into three ranges. A negative correlation between serum zinc concentration and serum copper concentration is seen in a serum creatinine level of <11.5 mg/dL (*r* = −0.394, *p* < 0.001) but not in the level of ≥11.5 mg/dL (*r* = 0.151, *p* = 0.104). (b) With regression lines reflecting serum transthyretin levels stratified into three ranges. A negative correlation between serum zinc and serum copper concentrations is seen in a serum transthyretin level of <30 mg/dL (*r* = −0.419, *p* < 0.001) but not in the level of ≥30 mg/dL (*r* = 0.140, *p* = 0.122).

Significant correlations were found between age, serum copper, serum creatinine, serum potassium, C-reactive protein (CRP), urea nitrogen, body weight, and diastolic blood pressure (all *p* < 0.001) (Table 3). Furthermore, serum copper concentration negatively correlated with serum zinc concentration. However, this negative correlation was not seen in patients with serum creatinine levels ≥11.5 mg/dL (Figure 6(a)). Similarly, there is no negative correlation between serum zinc and copper concentrations in patients with serum transthyretin levels ≥30 mg/dL (Figure 6(b)).

## 4. Discussion

Herein, two zinc formulations, zinc acetate hydrate and polaprezinc, were administered to HD patients with hypozincemia to compare the effects of zinc supplementation over 1 year.

Zinc is a typical essential trace element, along with copper (Cu) and selenium (Se), which act as metabolic catalysts. Growth retardation, poor wound healing, decreased sexual performance, hair and nail abnormalities, dysgeusia, gastrointestinal disorders (e.g., anorexia, abdominal pain, nausea, and glossitis), and abnormalities in neurobehavioral functions (e.g., increased anxiety and depression) result from zinc deficiency [1]. According to the “Overview of the Dietary Reference Intakes for Japanese (2020),” the recommended amount of zinc intake is 10–11 mg/day for men and 8 mg/day for women, and the upper limit to prevent overdose is 40–45 mg/day for men and 30–35 mg/day for women [6].

Excess zinc can cause rough hair, diarrhea, microcytic anemia, and hypercholesterolemia. Excess oral zinc intake may also lead to copper, manganese, and iron deficiencies because zinc competes with these elements for intestinal absorption. Zinc deficiency usually occurs more commonly than excess zinc in people with chronic disease such as kidney failure, kidney transplantation, anorexia, malnutrition, and older adults [1]. Zinc deficiency is diagnosed based on clinical symptoms, low alkaline phosphatase levels, a zinc-dependent enzyme, and serum zinc levels [2].

This study provided zinc supplementation for maintenance HD patients with low zinc levels of <60 *μ*g/dL. Supplementation was provided using two types of formulations clinically available in Japan, following the package insert of each drug. The zinc dose administered was higher in the ZAH group than in the PPZ group. In both the ZAH and PPZ groups, the mean serum zinc levels significantly increased from baseline to all observation points from 4 to 52 weeks. It increased more rapidly in the ZAH group than in the PPZ group until week 12, after which there were no significant differences between the two groups. The frequency of adverse events requiring reduction or discontinuation of zinc supplementation was higher in the ZAH group than in the PPZ group. Consequently, the mean administered zinc dose was significantly reduced in the ZAH group after 20 weeks.

Okamoto et al. reported zinc supplementation with ZAH and PPZ in HD patients [5]. This study used the same drugs and dosages employed in their study. However, the observation period and timing of the serum zinc and copper concentrations differed between the two studies. In a study by Okamoto et al., the observation period was 6 months and zinc and copper concentrations were measured at 1, 3, and 6 months after the commencement of zinc supplementation. Herein, the observation period was 52 weeks, and zinc and copper levels were monitored every 4 weeks from the start of zinc supplementation. Okamoto et al. concluded that ZAH was more effective than PPZ in terms of zinc concentration and achievement rates within the observation period. Our study found no significant difference between ZAH and PPZ in terms of zinc concentration and zinc concentration achievement rates after 16 weeks (Figure 2). ZAH had a higher rate of dose change due to adverse events (Figure 3). Our study found that ZAH can be replenished earlier than PPZ but requires more frequent concentration monitoring and dose reduction. PPZ requires longer replenishment but less dose reduction due to adverse events. Based on these characteristics, it is possible to use both types properly. Furthermore, in another study by Okamoto et al., the predialysis creatinine level was independently associated with responsiveness to zinc supplementation after 3 months [7]. Our study also found a significant negative correlation between the change in zinc concentration after 3 months and predialysis serum creatinine levels (*r* = −0.354, *p* = 0.003).

The absorption rate of zinc in humans is related to the amount of oral zinc intake, which ranges from 16% to 50% and increases to 92% in the presence of zinc deficiency. In the case of excess Zn, its excretion from the intestinal tract exceeds its absorption to maintain homeostasis [8]. Therefore, increasing the zinc dose did not lead to a corresponding increase in serum zinc levels.

Wang et al. reported that in maintenance HD patients, serum zinc levels after zinc supplementation did not correlate with baseline zinc levels or the supplemented zinc dose but correlated positively with the duration of the intervention period [4]. According to Kodama et al., zinc replacement therapy is not immediately effective, and the longer the treatment period, the stronger the effect [9]. Tonelli reported that zinc levels at 90 and 180 days after the start of supplementation were significantly higher in the supplementation group than in the nonsupplementation group; however, this effect was only observed with medium daily doses (50 mg/day) and not with low zinc doses (25 mg/day) [10]. According to the European Best Practice Guidelines on Nutrition, HD patients with chronically inadequate protein/energy intake and zinc deficiency symptoms should receive supplementation with 50 mg of elemental zinc daily for 3–6 months [11].

In this study, which involved a longer observation period with zinc supplementation than the previous reports mentioned above, we compared ZAH and PPZ as zinc supplements and identified the advantages of each. The results showed that receiving 1 tablet of PPZ twice daily (a zinc amount of 34 mg/day) was preferable because this formulation maintained serum zinc levels more stable than ZAH for 1 year. Meanwhile, ZAH is likely more effective when rapid increases in serum zinc levels are required to relieve symptoms of zinc deficiency. However, the ZAH dose should be adjusted with particular attention to the potential occurrence of copper deficiency.

Zinc supplementation increases serum zinc concentration and improves various nutritional indicators and inflammatory parameters [3, 12, 13]. In our study, despite no significant changes in nutritional and inflammatory indicators, serum zinc and copper concentrations changed, showing significant increases in both ZAH and PPZ groups throughout the observation period. A negative correlation was found between all the serum and CRP levels measured in this study, suggesting that zinc supplementation may suppress inflammation.

Regarding correlation with nutritional indicators, serum zinc concentration was negatively correlated with transthyretin and body weight but not with total protein or albumin levels. Although serum zinc levels were negatively correlated with serum copper levels in our study, the association between elevated serum zinc levels and nutritional indicators remains unclear. However, when the patients were stratified according to serum creatinine and transthyretin levels, no negative correlation was observed in the group with creatinine levels ≥11.5 mg/dL and in the group with transthyretin levels ≥30 mg/dL. This suggests that patients with good nutrition or high muscle mass are less likely to develop copper deficiency, even with increased zinc levels. Specifically, physicians should pay attention to the potential decrease in serum copper concentrations in HD patients who receive zinc supplementation, especially in those whose creatinine and transthyretin levels are low, normal, or not much higher than normal.

The question may arise as to whether it is better to supplement zinc at low doses for a long period or at high doses for a short period. Baarz et al. reported that zinc supplementation for six days in older patients with zinc-deficient increased serum zinc levels and suppressed interleukin-2 expression, suggesting improved immune function [14]. Their results highlighted the importance of identifying individuals who may benefit from immediate zinc supplementation, such as those with acute infections. The relationship between zinc and coronavirus disease 2019 (COVID-19), including how zinc deficiency affects COVID-19 severity and whether zinc supplementation improves clinical outcomes, is under investigation. The National Institutes of Health (NIH) recommends zinc supplementation above the recommended dietary allowance to prevent COVID-19 [15].

Although each case must be carefully considered, the NIH, European Food Safety Authority (EFSA), and other health authorities recommend that long-term low-dose zinc is more beneficial than short-term high-dose administration [16–18]. The NIH warns that prolonged denture adhesive creams containing up to 34 mg of zinc per gram of product can lead to neurological symptoms and anemia [15]. The EFSA warns that the long-term intake of zinc supplements >25 mg/day may lead to anemia [16]. Long-term zinc supplementation can cause copper deficiency, leading to reversible hematological defects (anemia and leukopenia) and potentially irreversible neurological manifestations (myelopathy, paresthesia, ataxia, and spasticity). Zinc supplementation should generally be maintained within tolerable upper intake limits, and serum copper levels should be measured.

Hypozincemia is also associated with anemia. Sato et al. reported that supplementation with 50 mg zinc acetate (reduced to 25 mg as appropriate) in HD patients with hypozincemia increased serum zinc concentration and significantly decreased ESA dosage and ERI, indicating that the resolution of hypozincemia contributed to reducing renal anemia [19]. Herein, the ZAH group maintained higher Hb levels with lower ESA doses and lower ERIs than the PPZ group throughout the observation period. This may be because the ZAH group maintained higher serum zinc concentrations than the PPZ group.

Copper deficiency due to excessive zinc supplementation has been reported to cause anemia and leukopenia. Marumo et al. reported the case of an 89-year-old man who developed pancytopenia after overdosing a zinc formulation for ESA-resistant anemia during maintenance HD [20]. In our study, no anemia or decrease in white blood cell, lymphocyte, or monocyte counts was observed during zinc supplementation, and there were no significant differences in the degree of change between the ZAH and PPZ groups. Copper deficiency requiring a reduction in the zinc dose occurred significantly more frequently in the ZAH group; however, appropriate dose reduction of ZAH prevented the development of anemia or decreased leukopenia.

This study had some limitations. First, the sample size was small, and the population comprised many older patients. Future studies should conduct a long-term observation and increase the number of patients.

## 5. Conclusions

In maintenance HD patients with zinc deficiency, daily administration of 2 tablets of PPZ (zinc amount: 34 mg) can safely maintain serum zinc levels for 1 year. Meanwhile, the daily administration of 2 tablets of ZAH (zinc amount: 50 mg) is effective in rapidly increasing serum zinc levels. However, it has a greater risk of adverse events such as copper deficiency, which requires zinc dose reduction or discontinuation. In clinical practice, zinc supplementation with these two drugs should be individualized according to the patient's medical condition.

## Figures and Tables

**Table 1:** Comparison of baseline patient characteristics by groups.

	ZAH group		PPZ group		*p* value
Number of patients	37		38		—
Age^a^ (years)	74.7	(12.8)	72.6	(13.0)	0.452
HD vintage^a^ (years)	7.2	(5.4)	7.5	(5.7)	0.860
Male sex^b^	28	(75.7%)	27	(71.1%)	0.651
Primary disease^b^					0.686
Chronic glomerulonephritis *n* (%)	15	(40.5%)	14	(36.8%)	
Diabetic nephropathy *n* (%)	16	(43.2%)	12	(31.6%)	
Other, *n* (%)	6	(16.3%)	12	(31.6%)	
Serum zinc^a^ (*μ*g/dL)	52.3	(4.9)	52.4	(5.2)	0.903
Serum copper^a^ (*μ*g/dL)	89.1	(15.3)	93.5	(17.0)	0.396
Alkaline phosphatase^a^ (U/L)	84.8	(30.2)	83.5	(34.1)	0.611
ALT^a^ (U/L)	11.3	(5.1)	12.3	(5.7)	0.517
AST^a^ (U/L)	13.4	(4.4)	14.8	(9.4)	0.983
LDH^a^ (U/L)	182.3	(37.2)	176.5	(43.9)	0.474
Total bilirubin^a^ (mg/dL)	0.37	(0.12)	0.37	(0.12)	0.772
Serum calcium^a^ (mg/dL)	8.67	(0.56)	8.79	(0.65)	0.176
Serum creatinine^a^ (mg/dL)	9.11	(2.51)	9.69	(2.62)	0.539
Serum chlorine^a^ (mEq/L)	101.8	(3.7)	102.3	(3.4)	0.516
Serum phosphate^a^ (mg/dL)	5.3	(1.0)	5.5	(1.4)	0.592
Serum sodium^a^ (mEq/L)	139.2	(3.1)	138.5	(3.4)	0.465
Serum potassium^a^ (mEq/L)	4.5	(0.6)	4.5	(0.6)	0.874
Hemoglobin^a^ (g/dL)	11.3	(1.1)	11.2	(1.1)	0.286
Ferritin^a^ (ng/mL)	184.6	(130.9)	171.3	(118.9)	0.798
TSAT^a^ (%)	25.6	(10.7)	27.5	(17.1)	0.567
Glucose^a^ (mg/dL)	155.4	(53.8)	141.1	(60.8)	0.075
LDL-cholesterol^a^ (mg/dL)	82.3	(18.7)	83.5	(20.1)	0.715
CRP^a^ (mg/dL)	0.99	(1.98)	0.80	(1.20)	0.824
Total cholesterol^a^ (mg/dL)	141.6	(23.2)	151.8	(25.4)	0.063
Total protein^a^ (g/dL)	6.1	(0.5)	6.3	(0.5)	0.307
Serum albumin^a^ (g/dL)	3.38	(0.26)	3.39	(0.32)	0.932
Transthyretin^a^ (mg/dL)	24.7	(5.5)	23.9	(5.5)	0.481
Urea nitrogen^a^ (mg/dL)	57.9	(13.1)	58.5	(14.0)	0.849
BNP^a^ (pg/mL)	299.1	(241.5)	329.4	(514.0)	0.322
Treat time^a^ (hours)	4.7	(0.6)	4.8	(0.6)	0.751
Dry body weight^a^ (kg)	57.9	(11.3)	56.5	(9.0)	0.656
Systolic blood pressure^a^ (mmHg)	149.9	(21.7)	150.4	(22.7)	0.870
Diastolic blood pressure^a^ (mmHg)	80.5	(15.1)	82.0	(12.6)	0.508

^a^Wilcoxon test. ^b^*χ*^2^ test. Data are presented as *n* (%) or as the mean (SD). ALT, alkaline aminotransferase; AST, aspartate aminotransferase; BNP, human brain natriuretic peptide; CRP, C-reactive protein; LDH, lactate dehydrogenase; TSAT, transferrin saturation.

**Table 2:** Adverse events and reasons for discontinuation or dose reduction of oral zinc supplementation.

	ZAH group		PPZ group		Total		*p* value
*(A) Within 24 weeks*							
Serum zinc level ≥130 *μ*g/dL	8	(21.6%)	3	(7.9%)	11	(14.7%)	0.093
Serum copper level <30 *μ*g/dL	4	(10.8%)	2	(5.3%)	6	(8.0%)	0.376
Gastrointestinal-related symptoms	23	(62.2%)	16	(42.1%)	39	(52.0%)	0.082
Blood-related symptoms	7	(18.9%)	4	(10.5%)	11	(14.7%)	0.304
Skin-related symptoms	2	(5.4%)	2	(5.3%)	4	(5.3%)	0.978
Other symptoms	1	(2.7%)	2	(5.3%)	3	(4.0%)	0.572

*(B) Within 52 weeks*							
Serum zinc level ≥130 *μ*g/dL	8	(21.6%)	3	(7.9%)	11	(14.7%)	0.093
Serum copper level <30 *μ*g/dL	5	(13.5%)	3	(7.9%)	8	(10.7%)	0.431
Gastrointestinal-related symptoms	23	(62.2%)	16	(42.1%)	39	(52.0%)	0.082
Blood-related symptoms	7	(18.9%)	4	(10.5%)	11	(14.7%)	0.304
Skin-related symptoms	2	(5.4%)	2	(5.3%)	4	(5.3%)	0.978
Other symptoms	2	(5.4%)	2	(5.3%)	4	(5.3%)	0.978

Data are presented as *n* (%).

**Table 3:** Coefficient of correlation of parameters with serum zinc concentration.

Parameters	Correlation coefficients	*p* value
Age	0.226	<0.0001
HD vintage	−0.143	0.007
Serum copper	−0.310	<0.0001
Alkaline phosphatase	0.085	0.112
ALT	−0.013	0.812
AST	0.033	0.537
LDH	0.047	0.379
Total bilirubin	0.023	0.673
Serum calcium	0.078	0.144
Serum creatinine	−0.284	<0.0001
Serum chlorine	−0.044	0.413
Serum phosphate	−0.076	0.153
Serum sodium	−0.009	0.872
Serum potassium	−0.209	<0.0001
Hemoglobin	0.009	0.864
Glucose	0.014	0.790
LDL-cholesterol	0.044	0.411
CRP	−0.141	0.008
Total cholesterol	−0.028	0.595
Total protein	0.060	0.257
Serum albumin	0.061	0.249
Transthyretin	−0.044	0.414
Urea nitrogen	−0.173	0.001
BNP	0.159	0.003
Dry body weight	−0.253	<0.0001
Systolic blood pressure	0.019	0.717
Diastolic blood pressure	−0.113	0.033

ALT, alkaline aminotransferase; AST, aspartate aminotransferase; BNP, brain natriuretic peptide; CRP, C-reactive protein; LDH, lactate dehydrogenase.

## Data Availability

The data used to support the findings of this study are available from the corresponding author upon request.

## Supplementary Materials

Supplementary Materials Supplementary file: changes in parameters in the study.
