# Supplementary material for: Zinc Acetate Hydrate Supplementation versus Polaprezinc Supplementation for Improving Hypozincemia in Hemodialysis Patients: A Randomized Clinical Trial
Source: Int J Nephrol. 2023 Oct 5;2023:2403755. doi: 10.1155/2023/2403755 (PMC10569889; doi:10.1155/2023/2403755)
Supplement: Supplementary Materials — Supplementary file: changes in parameters in the study. [file 2403755.f1.pdf]

**Supplementary material** Changes in Parameters in the Study.

| ZAH group | | Baseline | 4 week | 8 week | 12 week | 16 week | 20 week | 24 week | 52 week | *P* value |
|---|---|---|---|---|---|---|---|---|---|---|
| Serum zinc[a] (μg/dL) | Mean | 52.3 | 77.5 | 91.3 | 91.2 | 89.0 | 88.9 | 86.5 | 83.4 | <.001 |
| | (SD) | (4.9) | (21.4) | (24.3) | (26.3) | (26.5) | (22.7) | (28.3) | (19.9) | |
| Serum copper[a] (μg/dL) | Mean | 89.1 | 90.4 | 84.5 | 75.7 | 70.6 | 69.5 | 74.5 | 83.6 | .003 |
| | (SD) | (15.3) | (18.3) | (17.3) | (25.3) | (27.3) | (28.5) | (29.4) | (24.3) | |
| Alkaline phosphatase[a] (U/L) | Mean | 84.8 | 88.2 | 94.8 | 91.3 | 88.6 | 90.0 | 93.2 | 80.6 | .717 |
| | (SD) | (30.2) | (28.2) | (31.2) | (30.7) | (27.8) | (28.0) | (33.8) | (28.6) | |
| ALT[a] (U/L) | Mean | 11.3 | 12.5 | 13.5 | 12.3 | 12.9 | 12.0 | 12.4 | 14.3 | .819 |
| | (SD) | (5.1) | (5.6) | (5.8) | (5.7) | (5.9) | (6.0) | (5.2) | (7.9) | |
| AST[a] (U/L) | Mean | 13.4 | 14.0 | 14.7 | 13.8 | 13.7 | 13.4 | 13.2 | 16.9 | .985 |
| | (SD) | (4.4) | (5.5) | (6.6) | (4.5) | (4.5) | (4.5) | (4.7) | (10.6) | |
| LDH[a] (U/L) | Mean | 182.3 | 185.6 | 187.6 | 188.2 | 191.5 | 186.9 | 192.4 | 204.9 | .431 |
| | (SD) | (37.2) | (38.6) | (31.3) | (26.5) | (37.2) | (30.5) | (38.5) | (36.2) | |
| Total bilirubins[a] (mg/dL) | Mean | 0.37 | 0.37 | 0.41 | 0.40 | 0.42 | 0.41 | 0.42 | 0.42 | .743 |
| | (SD) | (0.12) | (0.12) | (0.13) | (0.16) | (0.16) | (0.17) | (0.19) | (0.13) | |
| Serum Calcium[a] (mg/dL) | Mean | 8.67 | 8.61 | 8.77 | 8.55 | 8.82 | 8.89 | 8.91 | 8.85 | .439 |
| | (SD) | (0.56) | (0.50) | (0.69) | (0.53) | (0.70) | (0.89) | (0.66) | (0.66) | |
| Serum creatinine[a] (mg/dL) | Mean | 9.11 | 9.16 | 8.93 | 8.79 | 8.86 | 8.92 | 9.05 | 8.72 | 1.000 |
| | (SD) | (2.51) | (2.62) | (2.68) | (2.66) | (2.78) | (2.85) | (2.91) | (2.95) | |
| Serum chlorine[a] (mEq/L) | Mean | 101.8 | 102.1 | 103.2 | 102.8 | 102.3 | 102.0 | 101.7 | 103.1 | .424 |
| | (SD) | (3.7) | (3.6) | (3.8) | (3.1) | (3.2) | (3.2) | (3.2) | (3.5) | |
| Serum phosphate[a] (mg/dL) | Mean | 5.3 | 5.1 | 5.1 | 5.1 | 5.1 | 5.0 | 5.0 | 5.2 | .949 |
| | (SD) | (1.0) | (1.1) | (1.2) | (1.5) | (1.3) | (1.1) | (1.0) | (1.2) | |
| Serum sodium[a] (mEq/L) | Mean | 139.2 | 139.2 | 140.2 | 139.6 | 139.8 | 139.5 | 138.9 | 139.7 | .711 |
| | (SD) | (3.1) | (3.1) | (2.6) | (2.8) | (2.3) | (3.0) | (2.9) | (3.0) | |
| Serum potassium[a] (mEq/L) | Mean | 4.5 | 4.6 | 4.4 | 4.4 | 4.5 | 4.5 | 4.5 | 4.4 | .992 |
| | (SD) | (0.6) | (0.8) | (0.5) | (0.6) | (0.7) | (0.7) | (0.5) | (0.5) | |
| Hemoglobin[a] (g/dL) | Mean | 11.3 | 11.4 | 11.3 | 11.2 | 11.1 | 11.3 | 11.7 | 11.3 | .413 |
| | (SD) | (1.1) | (1.1) | (0.9) | (1.0) | (1.0) | (1.0) | (1.0) | (1.2) | |
| Glucose[a] (mg/dL) | Mean | 155.4 | 154.3 | 154.2 | 145.7 | 152.4 | 147.3 | 140.0 | 148.1 | .865 |
| | (SD) | (53.8) | (67.9) | (48.8) | (52.3) | (52.4) | (50.1) | (51.5) | (46.5) | |
| LDL-cholesterol[a] (mg/dL) | Mean | 82.3 | 79.3 | 82.6 | 84.4 | 84.4 | 85.7 | 83.3 | 87.3 | .760 |
| | (SD) | (18.7) | (16.4) | (18.2) | (20.2) | (21.2) | (18.1) | (24.5) | (14.9) | |
| CRP[a] (mg/dL) | Mean | 0.99 | 1.52 | 0.69 | 0.70 | 1.06 | 0.39 | 2.08 | 0.56 | .750 |
| | (SD) | (1.98) | (3.53) | (0.70) | (1.70) | (2.97) | (0.44) | (6.39) | (1.25) | |
| Total cholesterol[a] (mg/dL) | Mean | 141.6 | 140.9 | 142.9 | 147.2 | 144.2 | 142.7 | 144.6 | 147.9 | .901 |
| | (SD) | (23.2) | (20.6) | (19.6) | (20.9) | (25.2) | (23.3) | (29.8) | (15.5) | |
| Total protein[a] (g/dL) | Mean | 6.1 | 6.1 | 6.2 | 6.2 | 6.1 | 6.1 | 6.2 | 6.2 | .969 |
| | (SD) | (0.5) | (0.5) | (0.5) | (0.4) | (0.4) | (0.4) | (0.4) | (0.5) | |
| Serum albumin[a] (g/dL) | Mean | 3.38 | 3.37 | 3.47 | 3.45 | 3.46 | 3.46 | 3.52 | 3.54 | .409 |
| | (SD) | (0.26) | (0.30) | (0.31) | (0.28) | (0.29) | (0.29) | (0.38) | (0.33) | |
| Transthyretin[a] (mg/dL) | Mean | 24.7 | 27.0 | 25.0 | 27.8 | 25.9 | 27.0 | 26.9 | 28.7 | .502 |
| | (SD) | (5.5) | (6.7) | (4.3) | (5.6) | (6.4) | (7.3) | (7.4) | (8.1) | |
| Urea nitrogen[a] (mg/dL) | Mean | 57.9 | 58.0 | 57.3 | 55.3 | 54.6 | 50.9 | 54.9 | 54.0 | .591 |
| | (SD) | (13.1) | (13.4) | (13.5) | (14.4) | (13.1) | (14.6) | (12.1) | (13.1) | |
| BNP[a] (pg/mL) | Mean | 299.1 | 305.7 | 301.7 | 322.5 | 335.8 | 314.9 | 234.8 | 389.8 | .665 |
| | (SD) | (241.5) | (250.4) | (260.1) | (303.2) | (270.0) | (319.8) | (244.7) | (306.3) | |
| Dry body weight[a] (kg) | Mean | 57.9 | 58.3 | 57.9 | 57.4 | 57.3 | 57.4 | 58.2 | 59.2 | 1.000 |
| | (SD) | (11.3) | (11.5) | (11.8) | (12.5) | (12.5) | (12.6) | (12.6) | (14.5) | |
| Systolic blood pressure[a] (mmHg) | Mean | 149.9 | 150.3 | 156.8 | 157.5 | 157.6 | 156.8 | 152.4 | 156.8 | .784 |
| | (SD) | (21.7) | (26.3) | (24.0) | (27.2) | (28.3) | (19.0) | (25.0) | (28.3) | |
| Diastolic blood pressure[a] (mmHg) | Mean | 80.5 | 80.9 | 80.2 | 81.8 | 83.5 | 80.2 | 82.3 | 80.5 | .965 |
| | (SD) | (15.1) | (14.0) | (13.5) | (11.7) | (15.8) | (13.7) | (11.5) | (16.7) | |

| PPZ group | | Baseline | 4 week | 8 week | 12 week | 16 week | 20 week | 24 week | 52 week | *P* value |
|---|---|---|---|---|---|---|---|---|---|---|
| Serum zinc[a] (μg/dL) | Mean | 52.4 | 68.1 | 77.8 | 77.4 | 79.5 | 77.4 | 80.8 | 79.9 | <.001 |
| | (SD) | (5.2) | (14.8) | (22.4) | (20.7) | (18.0) | (22.7) | (20.2) | (16.8) | |
| Serum copper[a] (μg/dL) | Mean | 93.5 | 93.4 | 86.3 | 82.8 | 79.6 | 80.4 | 82.9 | 80.0 | .016 |
| | (SD) | (17.0) | (18.6) | (17.8) | (22.8) | (23.2) | (19.7) | (17.6) | (25.4) | |
| Alkaline phosphatase[a] (U/L) | Mean | 83.5 | 91.4 | 87.6 | 90.7 | 96.8 | 86.8 | 87.0 | 86.2 | .998 |
| | (SD) | (34.1) | (43.7) | (36.8) | (44.2) | (54.7) | (40.9) | (36.1) | (37.7) | |
| ALT[a] (U/L) | Mean | 12.3 | 14.2 | 12.1 | 13.1 | 14.3 | 12.3 | 11.8 | 13.7 | .949 |
| | (SD) | (5.7) | (8.2) | (7.1) | (8.2) | (10.6) | (6.6) | (5.9) | (7.7) | |
| AST[a] (U/L) | Mean | 14.8 | 15.1 | 13.7 | 13.7 | 15.5 | 13.5 | 13.2 | 14.9 | .971 |
| | (SD) | (9.4) | (8.1) | (6.8) | (6.4) | (9.5) | (5.7) | (6.1) | (6.9) | |
| LDH[a] (U/L) | Mean | 176.5 | 181.9 | 178.1 | 172.4 | 172.4 | 170.0 | 175.3 | 189.5 | .920 |
| | (SD) | (43.9) | (48.5) | (43.8) | (30.6) | (35.3) | (33.4) | (37.3) | (52.0) | |
| Total bilirubins[a] (mg/dL) | Mean | 0.37 | 0.38 | 0.39 | 0.39 | 0.36 | 0.36 | 0.40 | 0.38 | .955 |
| | (SD) | (0.12) | (0.12) | (0.14) | (0.13) | (0.13) | (0.11) | (0.14) | (0.08) | |
| Serum Calcium[a] (mg/dL) | Mean | 8.79 | 8.69 | 8.61 | 8.62 | 8.58 | 8.67 | 8.75 | 8.64 | .827 |
| | (SD) | (0.65) | (0.52) | (0.35) | (0.50) | (0.56) | (0.48) | (0.56) | (0.50) | |
| Serum creatinine[a] (mg/dL) | Mean | 9.69 | 9.75 | 9.47 | 9.77 | 9.52 | 9.79 | 9.80 | 9.48 | .996 |
| | (SD) | (2.62) | (3.01) | (2.59) | (2.73) | (2.69) | (2.80) | (2.36) | (2.70) | |
| Serum chlorine[a] (mEq/L) | Mean | 102.3 | 101.4 | 102.4 | 102.2 | 101.4 | 102.0 | 102.2 | 102.0 | .875 |
| | (SD) | (3.4) | (2.9) | (2.7) | (3.4) | (3.1) | (3.3) | (2.9) | (3.8) | |
| Serum phosphate[a] (mg/dL) | Mean | 5.5 | 5.5 | 5.5 | 5.6 | 5.4 | 6.0 | 5.6 | 5.6 | .913 |
| | (SD) | (1.4) | (1.3) | (1.4) | (1.1) | (1.5) | (1.6) | (1.4) | (1.7) | |
| Serum sodium[a] (mEq/L) | Mean | 138.5 | 138.1 | 138.7 | 138.2 | 138.0 | 139.0 | 138.7 | 138.3 | .942 |
| | (SD) | (3.4) | (2.9) | (2.6) | (3.4) | (3.1) | (3.2) | (3.2) | (3.3) | |
| Serum potassium[a] (mEq/L) | Mean | 4.5 | 4.5 | 4.4 | 4.5 | 4.4 | 4.5 | 4.3 | 4.4 | .850 |
| | (SD) | (0.6) | (0.7) | (0.7) | (0.6) | (0.6) | (0.6) | (0.6) | (0.7) | |
| Hemoglobin[a] (g/dL) | Mean | 11.2 | 11.3 | 11.4 | 11.3 | 11.4 | 11.1 | 11.2 | 11.2 | .964 |
| | (SD) | (1.1) | (1.1) | (1.0) | (1.0) | (1.0) | (1.1) | (1.0) | (1.2) | |
| Glucose[a] (mg/dL) | Mean | 141.1 | 153.2 | 136.4 | 147.1 | 147.9 | 140.2 | 128.1 | 126.5 | .857 |
| | (SD) | (60.8) | (78.2) | (55.3) | (80.9) | (82.3) | (59.5) | (34.2) | (55.0) | |
| LDL-cholesterol[a] (mg/dL) | Mean | 83.5 | 83.4 | 84.3 | 82.0 | 81.0 | 81.7 | 85.0 | 84.3 | 1.000 |
| | (SD) | (20.1) | (21.0) | (19.4) | (18.2) | (21.7) | (20.2) | (24.8) | (22.7) | |
| CRP[a] (mg/dL) | Mean | 0.80 | 0.79 | 0.41 | 0.59 | 0.82 | 0.37 | 0.34 | 0.61 | .417 |
| | (SD) | (1.20) | (1.52) | (0.86) | (1.24) | (1.99) | (0.94) | (0.92) | (1.01) | |
| Total cholesterol[a] (mg/dL) | Mean | 151.8 | 149.8 | 149.9 | 147.8 | 145.3 | 145.5 | 150.5 | 149.2 | .998 |
| | (SD) | (25.4) | (25.0) | (23.8) | (24.0) | (29.1) | (25.6) | (33.1) | (27.8) | |
| Total protein[a] (g/dL) | Mean | 6.3 | 6.2 | 6.2 | 6.2 | 6.1 | 6.1 | 6.3 | 6.3 | .692 |
| | (SD) | (0.5) | (0.5) | (0.6) | (0.6) | (0.6) | (0.5) | (0.5) | (0.6) | |
| Serum albumin[a] (g/dL) | Mean | 3.39 | 3.40 | 3.46 | 3.43 | 3.42 | 3.46 | 3.52 | 3.49 | .711 |
| | (SD) | (0.32) | (0.43) | (0.34) | (0.37) | (0.40) | (0.36) | (0.34) | (0.48) | |
| Transthyretin[a] (mg/dL) | Mean | 23.9 | 25.8 | 24.6 | 26.9 | 25.6 | 26.5 | 26.6 | 28.1 | .794 |
| | (SD) | (5.5) | (7.8) | (6.6) | (6.9) | (8.0) | (7.2) | (7.0) | (9.8) | |
| Urea nitrogen[a] (mg/dL) | Mean | 58.5 | 63.3 | 56.8 | 62.3 | 59.4 | 60.5 | 58.5 | 62.6 | .651 |
| | (SD) | (14.0) | (18.1) | (14.6) | (15.5) | (17.4) | (16.3) | (15.5) | (15.8) | |
| BNP[a] (pg/mL) | Mean | 329.4 | 407.4 | 354.1 | 446.2 | 451.8 | 379.6 | 238.7 | 243.8 | .917 |
| | (SD) | (514.0) | (986.3) | (644.4) | (1130.2) | (1092.2) | (744.7) | (239.5) | (269.3) | |
| Dry body weight[a] (kg) | Mean | 56.5 | 56.4 | 55.8 | 55.6 | 55.1 | 55.4 | 55.9 | 55.5 | .998 |
| | (SD) | (9.0) | (9.2) | (9.3) | (9.4) | (9.6) | (9.8) | (9.5) | (8.9) | |
| Systolic blood pressure[a] (mmHg) | Mean | 150.4 | 151.8 | 150.6 | 149.8 | 147.9 | 147.3 | 147.6 | 151.9 | .989 |
| | (SD) | (22.7) | (21.6) | (31.0) | (24.4) | (22.8) | (22.2) | (19.1) | (17.6) | |
| Diastolic blood pressure[a] (mmHg) | Mean | 82.0 | 79.9 | 79.5 | 81.5 | 78.4 | 78.0 | 80.5 | 82.6 | .860 |
| | (SD) | (12.6) | (12.5) | (13.9) | (12.4) | (11.3) | (14.3) | (12.1) | (12.0) | |

[a] Wilcoxon test

Abbreviations: ALT, Alkaline aminotransferase; AST, Asparate aminotransferase; BNP, Human brain natriuretic peptide; CRP, C-relative protein; LDH, Lactate dehydrogenase.
